# Correlation between forkhead box P3 (rs3761548) gene polymorphism and serum interleukin13 as biomarkers of severity in Egyptian allergic conjunctivitis: a retrospective study

**DOI:** 10.3389/falgy.2024.1437600

**Published:** 2024-09-25

**Authors:** Wesam A. Boghdady, Marwa A. Khairy, Ali G. Ali, Alia A. El Shahawy, Eman A. Abdelaziz, Aya A. El Shahawy, Fatma Z. Kamel

**Affiliations:** ^1^ Department of Medical Microbiology and Immunology, Faculty of Medicine, Zagazig University, Zagazig, Egypt; ^2^ Department of Ophthalmology, Faculty of Medicine, Zagazig University, Zagazig, Egypt; ^3^ Medical Biochemistry and Molecular Biology Department, Faculty of Medicine, Zagazig University, Zagazig, Egypt; ^4^ Department of Clinical Pathology, Faculty of Medicine, Zagazig University, Zagazig, Egypt

**Keywords:** allergy, allergic conjunctivitis, forkhead box p3, polymorphism, interleukin 13, total immunoglobulin e, regulatory t cells

## Abstract

**Introduction:**

The genetic variants that alter human Forkhead Box P3 (*FOXP3*) function may have a part in the establishment of allergic conjunctivitis. Our study aimed to evaluate the *FOXP3* polymorphism, serum interleukin13 (IL13) and total immunoglobulin E (IgE) levels in allergic conjunctivitis and assess their role as biomarkers for allergic conjunctivitis risk and severity.

**Methods:**

This study included 52 cases and 52 controls. Blood samples were taken from allergic conjunctivitis patients and controls for total IgE, IL13 measurement and detection of *FOXP3* (rs3761548) gene polymorphism.

**Results:**

There was a statistically significant difference between the allergic conjunctivitis group and healthy control group regarding *FOXP3* (rs3761548) polymorphism with those have AA genotype are 12 times at risk for allergic conjunctivitis and A allele increases the risk of allergic conjunctivitis by about 4 times. There was statistically significant difference between mild/moderate and severe allergic conjunctivitis regarding *FOXP3* (rs3761548) polymorphism with those have AA genotype are 53 times at risk for severe allergic conjunctivitis and A allele increases the risk of severe allergic conjunctivitis by about 6 times. Also, there was a significantly higher value of total IgE IU/ml, IL13 Pg/ml value in severe allergic conjunctivitis compared to moderate/mild allergic conjunctivitis. The best cutoff values of total IgE and serum IL13 for detecting the severity of allergic conjunctivitis were ≥320 IU/ml and ≥40 Pg/ml and the area under the curve were 0.89 and 0.95 respectively.

**Conclusion:**

The research significantly contributes to find correlation of *FOXP3* polymorphism, total IgE and IL13 with risk and severity of allergic conjunctivitis which are limited in the literature on the perceived value relevance of *FOXP3* polymorphism in allergic conjunctivitis risk and severity.

## 1 Introduction

Allergic conjunctivitis is becoming more common in both adults and children. It significantly lowers the quality of life of those affected and can occasionally result in permanent vision loss (1). Allergic conjunctivitis is one of the most prevalent ocular diseases. The prevalence of allergic conjunctivitis has been rising recently, coinciding with environmental degradation, a rise in local haze, frequent use of less quality makeup, wearing contact lenses, and other factors (2). Allergic conjunctivitis is the only ocular disease to involve a type I hypersensitivity reaction. Th2 cells secrete pro-inflammatory cytokines [interleukin 3 (IL3), IL4, IL5, and IL13] in sensitized persons, which induce B cells to produce immunoglobulin E (IgE) (3).

The pleiotropic cytokine IL13 regulates the expression of proinflammatory cytokines by macrophages, overexpresses adhesion molecules, stimulates B-cell production of IgE, and influences mucus synthesis (4). Elevated cytokine concentrations, such as IL13, play a crucial proinflammatory role in ocular allergy through the stimulation of IgE synthesis (5). The Th2 phenotype is more common in allergic people, which causes an increase in IL13 production and class switching in B cells to produce IgE. Patients with allergic conjunctivitis had greater serum levels of IL13 (6).

The Forkhead Box P3 (*FOXP3*) gene is situated on the X human chromosome. CD4+ CD25+ *FOXP3+* regulatory T cells (Tregs) express constitutively the transcription factor *FOXP3* which plays a crucial role in immunological homeostasis maintenance. Additionally, *FOXP3* suppresses the Th2 response after allergen exposure (7). While *FOXP3*, a master regulator of T regulatory cells, is essential in allergic diseases, its effect on disease risk due to gene variants remains unclear (8).

The genetic variants that alter *FOXP3* function may have a part in the establishment of allergic conjunctivitis and other allergic diseases like allergic rhinitis and asthma. Polymorphisms in the *FOXP3 gene* were associated with the risk of atopy, allergic rhinitis and asthma development by activation and development of regulatory T cells (9–11). Hence *FOXP3* polymorphism may have role in allergic conjunctivitis severity (7). However, studies investigating the correlation of *FOXP3* polymorphism, serum IL13 and total IgE in allergic conjunctivitis are still lacking. To expore this an important topic, the aim of the study is to evaluate the *FOXP3* polymorphism, IL13 and total IgE levels in allergic conjunctivitis and assess their role as biomarkers for allergic conjunctivitis risk and severity.

## 2 Patients and methods

### 2.1 Sample size

As mean difference in total IgE between cases and control was 237.4 ± 138.71, 174.44 ± 79.89 respectively so sample size is 104 with 52 are cases and 52 controls. Samples were calculated using OpenEpi program with confidence interval 95% at power of test 80% (12).

### 2.2 Study participants

This single blinded case control observational study was performed from December 2023 to May 2024 at ophthalmology and allergy unit in Zagazig University Hospitals. The work was done under the Ethics’ code of the World Medical Association (Declaration of Helsinki) for experiments including human cases. The Institutional Review Board Committee of the faculty of Medicine, Zagazig University authorized the study protocol (approval No. 11368/6-12-2023). Written consent was collected from allergic conjunctivitis patients and healthy controls who were included in the study. Patients who meet the diagnostic guidelines of allergic conjunctivitis (13), high level of total IgE level and positive skin prick testing were included in our study. While patients with other allergic diseases as bronchial asthma, those undergoing chronic treatment with systemic steroids or B-blockers, chronic inflammatory condition as chronic hepatitis, aspergillosis and tuberculosis, malignancy, systemic immunological disorders as rheumatoid arthritis, systemic lupus erythematous, and systemic sclerosis, and ischemic heart diseases, were excluded from our study. This study included 52 cases and 52 controls. Medical history of patients includes clinical manifestations, history of atopy, clinical score of allergic conjunctivitis severity, total ocular symptoms score (TOSS), skin prick test for relevant aeroallergens. Evey possible comparison between the study groups was considered. Blood samples were taken from allergic conjunctivitis patients and controls in plain and EDTA tube and whole blood samples in the plain tube were centrifugated at 1,409 xg for 15 min (min) to isolate serum then the serum was stored at −20 °C for total IgE and IL13 measurement and whole blood samples in the EDTA tube were stored at −20 °C for *FOXP3* restriction enzyme polymorphism.

### 2.3 Total ocular symptoms score

The TOSS is calculated as the sum of the patients’ scoring of the 3 individual ocular symptoms “itching/burning, tearing/watering, and redness” on a 0–3 categorical severity scale (0 = absent, 1 = mild, 2 = moderate, 3 = severe) so after summation of three symptoms, mild <4, moderate from 4 to 7, severe from 7 to 9 (14).

### 2.4 Skin prick test for relevant aeroallergens

The skin prick test was done using standardized inmunotek aeroallergens “pollens (grass Mix and cereal Mix), house dust mites (dermatophagoides pteronyssinus and dermatophagoides farina), mixed fungi (aspergillus niger, aspergillus fumigatus, candida albicans, alternaria alternata, and Cladosporium), cat dander, dog dander, rabbit dander and sunflower seeds”. Prior to skin testing, subjects were instructed to stop corticosteroids for 14 days and antihistamines for 5 days. The aeroallergens were placed in forearm and then the skin was pricked using saline 0.9% as negative control and the positive control was histamine dihydrochloride (10 mg/ml). The wheel's greatest diameter was measured after 10 min; a measurement of 3 mm or more was considered positive (5).

### 2.5 Total serum IgE measurement

Total serum IgE was detected by sandwich ELISA commercially available kit (Biocheck, Biokit, South San Francisco, CA 94080) according to the manufacturer's instructions. After adding the serum samples, the IgE antibody-coated micro titer wells are incubated for 30 min at room temperature with zero buffer. IgE antibody labelled with horseradish peroxidase (conjugate) is then introduced to the well after it has been cleaned to eliminate any remaining test specimen. Following a 30 min incubation at room temperature, the wells were washed to get rid of unbound, labelled antibodies. After adding a tetramethylbenzidine reagent solution and letting it sit at room temperature for 20 min, a blue colour starts to appear. When stop solution is added, the colour development was changed, turned yellow, and was measured by spectrophotometer at 450 nm. The test sample's colour intensity is directly correlated with the IgE content. Serum total IgE levels were measured as IU/ml with a limit of sensitivity (5.0 IU/ml) (5).

### 2.6 Serum IL13 measurement

Serum IL13 level was detected by sandwich ELISA using (FINE TEST No. EH3266, Optics Valley Biomedical Industrial Park, Wuhan, China). The capture antibody was pre-coated onto 96-well plates and the biotin-conjugated antibody was utilized as the detection antibody. The test samples, biotin-conjugated detection antibody, and standards were subsequently added to the wells and cleaned using wash buffer. After adding horseradish peroxidase streptavidin, unbound conjugates were removed using clean the barrier. Tetramethylbenzidine substrates were employed to see the enzymatic horseradish peroxidase streptavidin response. Horseradish peroxidase streptavidin catalyzed tetramethylbenzidine to yield a blue-colored molecule that turned yellow upon the addition of an acidic stop solution. Serum IL13 levels were measured as Pg/ml with a limit of sensitivity (9.375 Pg/ml) (5).

### 2.7 *FOXP3* (rs3761548) gene polymorphism

#### 2.7.1 Genomic DNA extraction and detection of DNA purity

Genomic DNA was extracted using a genomic DNA extraction kit (GeneJET, Thermo Scientific, USA, Cat. no. K078). The purity of the extracted genomic DNA was measured using the Nanodrop 2000 (Thermofischer Scientific, Wilmingiton, DE, USA) at 260 nm absorbance with a ratio between (1.7 and 1.8) for A260/A280. The genomic DNA was stored at −20 °C.

#### 2.7.2 DNA amplification by PCR

*FOXP3* (rs3761548) gene polymorphism was analyzed using restriction fragment length polymorphism (RFLP) (15). Primers for PCR amplification for *rs3761548 FOXP3 gene* is: Forward primer: (5′-GCCCTTGTCTACTCCACGCCTCT-3′) Reverse primer: (5′-CAGCCTTCGCCAATACAGAGCC-3′).

Primers were designed to give a predicted product size of 487 bp. These primers were supplied by (Invitrogen, Thermofisher scientific, analysis, USA). For each reaction, the following materials were added to each tube: master mix 10 μl, 1 μl of each primer, 5 μl of template DNA and sterile distilled water 3 μl to a total volume of 20 μl. The thermal cycler was set up to carry out the initial denaturation stage for 5 min at 95 °C, then 35 cycles of denaturation for thirty seconds (s) at 95 °C, and 45 cycles of annealing at 60 °C. 45 s of extension at 72 °C, followed by the last extension at 72 °C for 5 min.

#### 2.7.3 Detection of the amplified products by gel electrophoresis

The rs3761548 FOXP3 gene gives fluorescent bands at 487 bp.

#### 2.7.4 RFLP for Foxp3 rs3761548 gene

The restriction enzyme used was PstI supplemented with NE Buffer (10X) (New England BioLabs, UK). Patients with homozygous mutant type AA genotype gave one fluorescent band at 487 bp. Patients with wild normal genotype CC genotype gave two fluorescent band 329 and 158 bp. Patients with heterozygous mutant type AC gave three fluorescent bands at 487 bp, 329 bp, 158 bp.

### 2.8 Statistical analysis

Hardy–Weinberg equilibrium was used to compute the genotype distribution. The normality of the quantitative variables was examined. When comparing quantitative variables, independent sample *t*-tests and ANOVA were used for parametric data, and the Mann-Whitney was used for nonparametric data. Yates correction was applied to the chi-square (*χ*^2^) test in order to analyze the genotype/allele frequencies. Odds rations (ORs) and 95% CIs were determined whenever *χ*^2^ or Fisher's exact test was significant. Spearman's correlation coefficient was used to evaluate the correlation between the total IgE level, the total ocular symptoms score, and the serum IL13 levels in allergic conjunctivitis patients. If *P* values were less than 0.05, they were regarded as statistically significant. The Statistical Package of Social Sciences (SPSS) program (version 22, Chicago, USA) was used to compute the results.

## 3 Results

### 3.1 Baselines characteristics of study groups

Our study involved 52 allergic conjunctivitis patients, A total of 30 females (57.7%) and 22 males (42.3%), the mean age in all allergic conjunctivitis patients was 13.90 ± 5.76 years and 52 controls with 27 females (51.9%) and 25 males (48.1%) and their mean age was 14.44 ± 5.45. There was not a significant difference between the allergic conjunctivitis patients and healthy controls regarding gender and age (*P* = 0.554, 0.546) as illustrated in Table 1. There was a highly statistically significant difference between allergic conjunctivitis patients and controls as regard total IgE levels with mean difference (355.96 ± 92.19 IU/ml and 46.39 ± 30.05 IU/ml) respectively, *P* = 0.0001 Table 1. There was a highly statistically significant difference between allergic conjunctivitis patients and controls as regard IL13 levels with mean difference (49.04 ± 21.12 Pg/ml and 4.79 ± 2.72 Pg/ml) respectively, *P* = 0.0001 Table 1. Allergic conjunctivitis was commonly associated with positive family history to atopy with percentage 57.7% as shown in Table 1. Also, allergic conjunctivitis was more common in rural than urban areas with a percentage of 63.5% as shown in Table 1. Pollens, house dust mite and mixed fungi were the most common aeroallergens among allergic conjunctivitis patients with percentage 84.6%, 46.2 and 19.2% respectively as indicated in Table 1.

**Table 1:** Basic characters of studied groups:.

Parameters	Allergic conjunctivitis group (*n* = 52)	Healthy control group (*n* = 52)	*χ*^2^^a^/*u*-test^b^	*P*-value
Gender *n* (%)				
Female	30 (57.7%)	27 (51.9%)	0.349^a^	0.554
Male	22 (42.3%)	25 (48.1%)		
Age per years				
Mean ± SD	13.90 ± 5.76	14.44 ± 5.45	0.603^b^	0.546
Median (range)	13 (6–25)	14 (7–24)		
Total IgE IU/ml			8.77^b^	
Mean ± SD	355.96 ± 92.19	46.39 ± 30.05		0.0001*
Median(range)	375 (130–500)	40 (15–150)		
IL13 Pg/ml				
Mean ± SD	49.04 ± 21.12	4.79 ± 2.72	8.79^b^	0.0001*
Median(range)	42.5 (18.34–90.4)	4.9 (1.02–9.6)		** **
Skin prick test results	*n*.%			
Pollen	44 84.6			
House dust mites	24 46.2			
Fungi	10 19.2			
Family history(positive)	30 57.7			
Residence(rural)	33 63.5			

n, number, SD, standard deviation

^a^ *χ*^2^ Chi-square test.

^b^ u: Mann–Whitney *u* test.

*P value <0.05*:* significant.

### 3.2 *FOXP3* polymorphism among the studied groups

**Figure 1:**
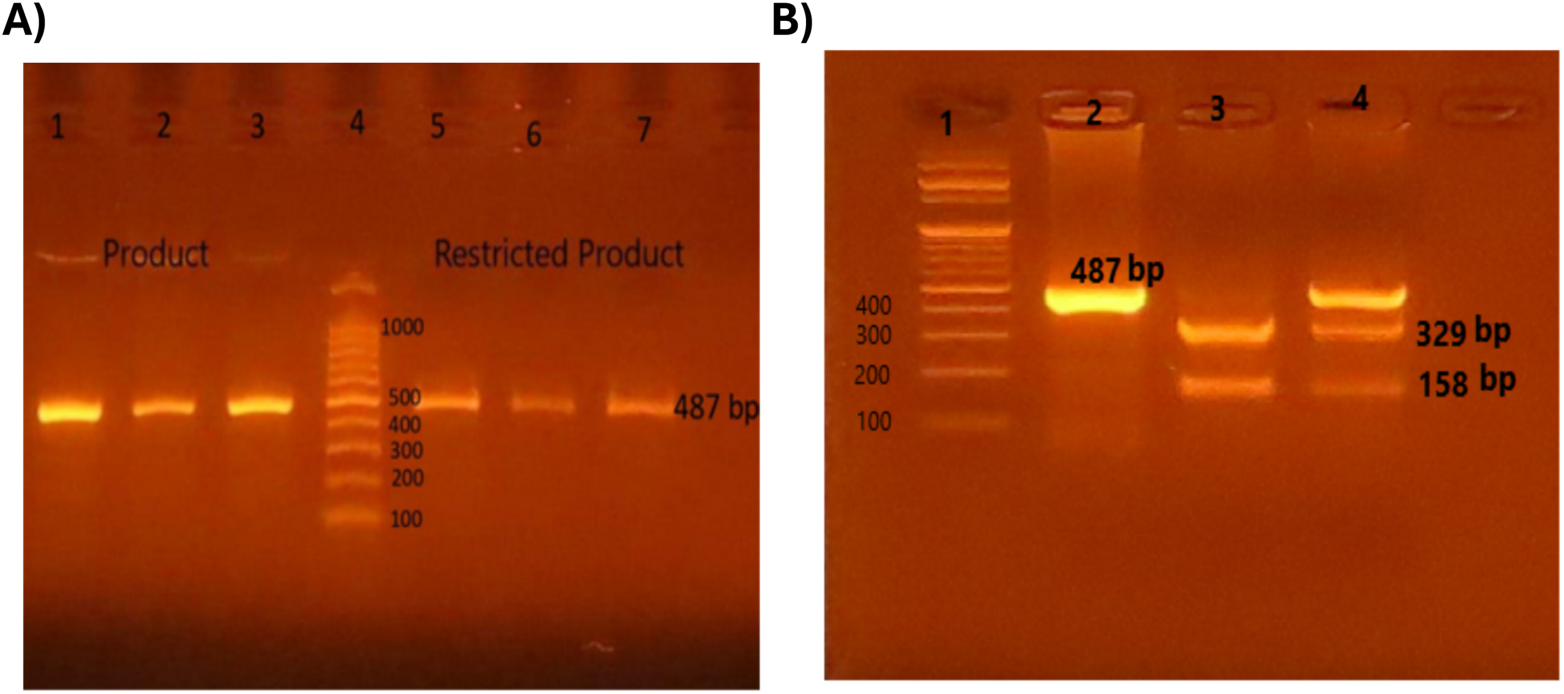
Detection of restriction fragment length polymorphism (RFLP) for Foxp3 rs3761548 gene by gel electrophoresis. Polymerase chain reaction restriction fragment length polymorphism (PCR-REFLP) pattern of 487 bp fragments product before and after digestion with **PstI** enzyme in order to detect (*rs3761548*) SNP in *FOXP3* gene. **(A) Lane 1, 2, 3: amplified product before PstI, Lane 4:** 100 bp DNA ladder, **Lane 3, 5, 6:** one band (487 bp) represent homozygous mutant type (AA). **(B) Lane 1:** 100 bp DNA ladder, **Lane 2:** one band (487 bp) represent homozygous mutant type (AA), **Lane 3:** two bands (329 bp & 158 bp) represent wild type (CC), **Lane 4:** three bands (487 bp, 329 bp & 158 bp) represent heterozygous mutant type (AC).

Genotypic frequencies of FOXP3 (rs3761548) gene were noticed in line with Hardy–Weinberg equilibrium in controls (*χ*^2^ = 2.47; *P* = 0.11) and cases (*χ*^2^ = 2.53; *P* = 0.11). There was statistically significant difference between the studied groups as regard *FOXP3* (rs3761548) polymorphism with those have AA genotype are 12.8 times at risk for allergic conjunctivitis, and A allele increases the risk of allergic conjunctivitis by about 4.6 times, *P* = 0.0001 Table 2. Considering the severity of allergic conjunctivitis, there was statistically significant difference between mild/moderate and severe allergic conjunctivitis regarding *FOXP3* (rs3761548) polymorphism with those have AA genotype are 53.3 times at risk for severe allergic conjunctivitis and A allele increases the risk of severe allergic conjunctivitis by about 6.8 times, *P* = 0.0001 as shown in Table 3 Fig. 1. There was a highly significant relation between TOSS and *FOXP3* gene polymorphism in allergic conjunctivitis patients, *p* = 0.0001 as displayed in Table 4.

**Table 2:** Comparison between *FOXP3* gene polymorphisms in allergic conjunctivitis patients and healthy controls.

FOXP3 gene	Allergic conjunctivitis (*n* = 52)	Healthy controls (*n* = 52)	*χ* ^2^ ^a^	*P*-value	OR (95% CI)
Genotype *n*, (%)		21.72	0.0001*		
AA	23 (44.2%)	6 (11.5%)	4.5	0.044*^b^	12.8 (3.98–41.05)
AC	20 (38.5%)	16 (30.8%)	21	0.0001*^c^	3.067 (1.007–9.336)
CC	9 (17.3%)	30 (57.7%)	8.3	0.004*^d^	4.167 (1.543–11.25)
Allele *n*, (%)					
A	66 (63.2%)	28 (45.2%)	28.03	0.0001*	4.6 (2.6–8.5)
C	38 (36.5%)	76 (73.1%)		ref	

n, number; OR, odds ratio; CI, confidence interval.

^a^ *χ*^2^ Chi square test of significant.

^b^ Compare AA&AC genotypes.

^c^ Compare AA& CC genotypes.

^d^ Compare AC &CC genotypes.

*P value <0.05: significant, ref (reference allele).

**Table 3:** Distribution of *FOXP3* gene polymorphisms in allergic conjunctivitis patients according to severity.

FOXP3 gene	Severe (*n* = 31)	Moderate/mild (*n* = 21)	*χ* ^2^ ^a^	*P*-value	OR (95% CI)
Genotype n, (%)		16.7	0.0001*		
AA	20 (64.5%)	3 (14.3%)	6.93	0.008*^b^	53.3 (4.8–592)
AC	10 (32.3%)	10 (47.6%)	f	0.0001*^c^	6.7 (1.5–29.8)
CC	1 (3.2%)	8 (38.1%)	f	0.096*^d^	8.0 (0.838–76.36)
Allele n, (%)					
A	50 (80.6%)	16 (38.1%)	19.5	0.0001*	6.8 (2.79–16.42)
C	12 (19.4%)	26 (61.9%)		ref	

n, number; OR, odds ratio; CI, confidence interval; f, fisher exact test.

^a^ *χ*^2^ Chi square test of significant.

^b^ Compare AA&AC genotypes.

^c^ Compare AA& CC genotypes.

^d^ Compare AC &CC genotypes.

*P value: <0.05: significant, ref (reference allele).

**Table 4:** Comparison between different *FOXP3* gene polymorphisms as regard total ocular symptoms score (TOSS) in allergic conjunctivitis cases.

Parameter	*FOX3* Gene Polymorphism			*f*	*P*-value	Tukey *post hoc*
	Homozygote mutant AA (*n* = 23)	Heterozygous AC (*n* = 20)	Homozygote wild CC (*n* = 9)			
Total ocular symptoms score (TOSS) Mean ± SD	7.96 ± 1.14	6.5 ± 1.31	3.67 ± 1.41	37.67	0.0001*	P1 0.004*a
						P2 0.0001*b
						P3 0.0001*c
TOSS Mean ± SD Median(range)	Allele A (*n* = 66)	Allele C (*n* = 38)		U 8.76	0.0001*	
	7.56 ± 1.32	5.2 ± 2				
	8 (7–9)	5 (3–6)				

n, number; SD, standard deviation; f, ANOVA test; *u*, Mann–Whitney Test.

*P value <0.05: significant.

**Figure 2:**
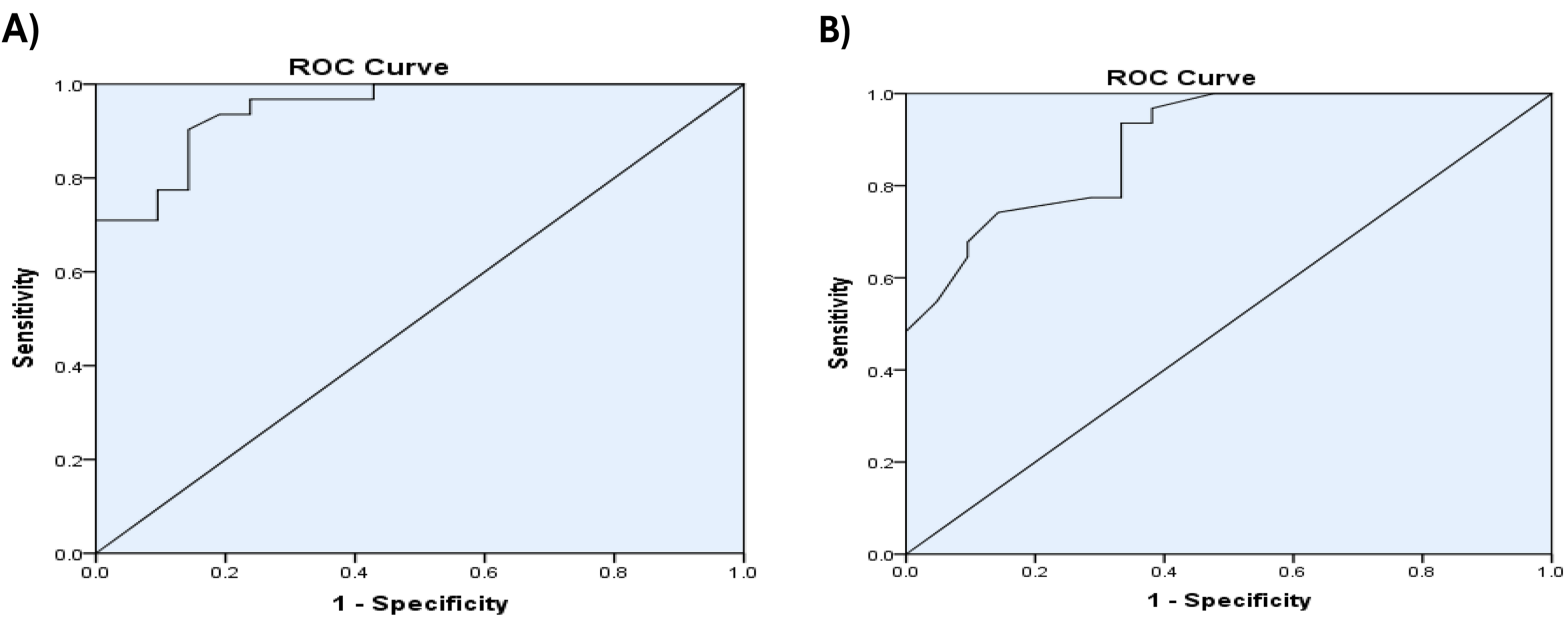
**(A)** Receiver operator characteristic curve (ROC) of IL13 Pg/ml in allergic conjunctivitis patients, **(B)** receiver operator characteristic curve (ROC) of total IgE IU/ml in allergic conjunctivitis patients.

There was a significant higher value of total IgE IU/ml with mean difference 407.10 ± 50.68 in severe allergic conjunctivitis compared to mean difference 280.48 ± 88.23 in moderate/mild allergic conjunctivitis, *p* < 0.05 as shown in Table 5. There was a significant higher value of serum IL13 levels pg/ml with mean difference 61.54 ± 17.39 in severe allergic conjunctivitis compared to mean difference 30.59 ± 9.19 in moderate/mild allergic conjunctivitis, *p* < 0.05 as shown in Table 5. Receiver operating characteristic curves analysis was carried out to analyze the total IgE, and IL13 as biomarkers for detecting the severity of allergic conjunctivitis in studied patients. The ROC curve of total IgE, IL13 showed that the level of total IgE at a cut-off value of ≥320 IUml and the area under the curve (AUC) is 0.89 with (95%CI: 0.81–0.978) (*p*-value 0.002) and the level of IL13 at a cut-off value of ≥40 Pg/ml and the AUC is 0.95 with (95%CI: 0.895–1) (*p*-value 0.003) as shown in Figure 2. This means that IL13 and total IgE are good biomarkers for detecting the severity of allergic conjunctivitis in studied patients. There was a significant strong positive correlation between total IgE IU/ml and IL13 Pg/ml, and a significant strong positive correlation between IL13 Pg/ml and TOSS with *p* = 0.0001 as shown in Table 6.

**Table 5:** Comparison of total IgE, IL13 levels according to severity allergic conjunctivitis.

Variables	Severity of allergic conjunctivitis		*t*	*P*-value
	Severe (*n* = 31)	Moderate/mild (*n* = 21)		
Total IgE IU/ml				
Mean ± SD	407.10 ± 50.68	280.48 ± 88.23		
median (range)	420 (300–500)	260 (130–420)	4.79	0.0001*
IL13 Pg/ml				
Mean ± SD	61.54 ± 17.39	30.59 ± 9.19		
median(range)	60.3 (30.10–90.4)	30 (18.34–52.38)	5.45	0.0001*

n, number; SD, standard deviation; *t*, student’ *t* test

*P value <0.05: significant.

**Table 6:** Correlation between total IgE IU/ml, IL13 Pg/ml, and total ocular symptoms score (TOSS) in allergic conjunctivitis patients (*n* = 52).

	Total IgE IU/ml		IL13 Pg/ml	
	*r*	*P*-value	*r*	*P*-value
IL13 Pg/ml	0.809**	0.0001*		
Total ocular symptoms score (TOSS)	0.681**	0.0001*	0.792**	0.0001*

Correlation coefficient (*r*), **Strong correlation. *P value <0.05: significant.

## 4 Discussion

Allergic conjunctivitis is an immunological hypersensitivity condition affecting the ocular conjunctiva mostly caused by an IgE mechanism (16). Up to 40% of adults suffer from allergic conjunctivitis, a very common condition brought on by the body's reaction to environmental allergens in the eyes (1). A person's genetic background and environmental factors, such as exposure to allergens, respiratory infections, and air pollution, can interact to cause allergic disorders. The transcription factor *FOXP3* is essential for immunological homeostasis maintenance and is constitutively expressed in CD4+ CD25+ FOXP3+ Tregs. Several studies showed that patients with allergy express lower FOXP3 expression than healthy controls. Thus, the etiology of additional allergy illnesses may involve in the impairment of *FOXP3* activity brought on by genetic polymorphisms and/or epigenetic mechanisms (7).

*FOXP3* is a particular marker and is essential to the growth and function of Tregs, which are cells that negatively regulate immunological responses (17). Several allergic disorders have been linked to variations in the *FOXP3* gene polymorphisms (18). There were several studies investigating the relation between *FOXP3* gene expression and polymorphism in allergic conditions, including allergic rhinitis (19) and allergic asthma (5). However, this gene in allergic conjunctivitis hasn't been studied yet. Increasing cytokine concentrations, such as IL13, play a crucial proinflammatory role in asthma and are typically coexisting with ocular allergies through the stimulation of IgE synthesis which cause mast cell degranulation and symptoms of allergy (5). Our study aimed to evaluate the *FOXP3* polymorphism, IL13 and total IgE levels in allergic conjunctivitis and assess their role as biomarkers for allergic conjunctivitis risk and severity.

The present study 30 females (57.7%) and 22 males (42.3%), the mean age in all patients was 13.90 ± 5.76 years and 52 controls with 27 females (51.9%) and 25 males (48.1%) and their mean age was 14.44 ± 5.45 with no significant difference between the studied groups regarding gender and age (*P* = 0.554, 0.546). Our results regarding the higher prevalence of allergic conjunctivitis in female patients is in agreement with Alqurashi et al. who showed that was significantly higher in females compared to males (20). Also, Yamana et al. showed that allergic conjunctivitis patients involved 33 (33.3%) male, and 66 (66.7%) female individuals aged 9–86 years (21).

In the current study, there was a highly statistically significant difference between allergic conjunctivitis patients and controls as regard total IgE, *P* = 0.0001. This is in agreement with Bao et al. who showed that the baseline concentrations of total IgE in patients with allergic conjunctivitis were significantly higher than those of control participants (*p* < 0.01) (22). Also, Yamana et al. showed that the total IgE test was positive in 68 (68.7%) allergic conjunctivitis patients (21). Regarding IL13, there was a highly statistically significant difference between allergic conjunctivitis patients and controls, *P* = 0.0001. This result agrees with Tao et al. who showed that serum IL13 was raised in allergic conjunctivitis patients compared to healthy individuals (23).

Our study showed that pollens, house dust mite and mixed fungi were the most common aeroallergens among allergic conjunctivitis patients with percentage 84.6%, 46.2 and 19.2% respectively. Abo-Ali et al. also showed similar results as the most prevalent aeroallergens among all patients were mites and pollens followed by hay dust (24). Also, Yamana et al. showed that pollen is the most common aeroallergens in allergic conjunctivitis patients (21).

Regarding *FOX3* gene polymorphisms as regard risk of allergic conjunctivitis, in this study, there was statistically significant difference between allergic conjunctivitis and healthy controls; with those have AA genotype are 12.8 times at risk for allergic conjunctivitis and A allele increases the risk of allergic conjunctivitis by about 4.6 times, *P* = 0.0001 that is agreed with Beigh et al. and Hassannia et al. They found that patients with *FOXP3* rs3761548 A allele had a significantly higher frequency of this haplotype than controls. So, A allele is a risk factor for the disease (8, 25). Also, this was in line with Marques et al. who stated that lower levels of FOXP3 gene expression in allergic conjunctivitis versus healthy controls. Consequently, the etiology of allergic disorders may include in the degradation of *FOXP3* function (7). However, our results disagreed with Yang et al. who showed that *Foxp3* polymorphisms did not differ between dust mite allergic conjunctivitis and healthy controls (26). This may be due to different population, genetic difference and the studied allergens was house dust mite but, in our studies, the most common allergens were pollens.

According to *FOXP3* polymorphism as regard severity, there was statistically significant difference between mild/moderate and severe allergic conjunctivitis regarding *FOXP3* (rs3761548) polymorphism; with those have AA genotype are 53.3 times at risk for severe allergic conjunctivitis and A allele increases the risk of severe allergic conjunctivitis by about 6 times, *P* = 0.0001. However, these results disagreed with Salah et al. who showed that genotypic distribution did not show a significant difference between mild, moderate and severe persistent allergic conjunctivitis (27). This may be explained by the different allergic diseases which were asthma, and our study included allergic conjunctivitis patients not asthmatic patients.

In concern with total IgE, there was a significantly higher value of total IgE IU/ml in severe allergic conjunctivitis compared to moderate/mild allergic conjunctivitis, *p* < 0.05 that agreed with Bao et al. who found that total IgE correlated with the severity of allergic conjunctivitis (22). Our study analyzed the total IgE as a biomarker for detecting severity in allergic conjunctivitis patients using ROC curve analysis and revealed that serum total IgE at a cut-off value of ≥320 IUml and AUC is 0.89 with (95%CI: 0.81–0.978) (*p*-value 0.002). This means that total IgE is a good biomarker for detecting the severity of allergic conjunctivitis in the studied patients. This was consistent with Roy et al. who showed that IgE is important in the immunopathology of allergic diseases and considered as an important marker. Serum IgE levels identified that a number of cytokines including IL13 with their role as biomarkers in allergic diseases (28). Also, this agrees with Yamana et al.*,* Bao et al. and Ezechukwu et al. who showed that total IgE correlated with severity of allergic conjunctivitis (21, 22, 29).

The present study analyzed serum IL13 as a biomarker for detecting the disease severity in allergic conjunctivitis patients using ROC curve analysis and revealed that serum IL13 at a cut-off value of ≥40 Pg/ml and the AUC is 0.95 with (95%CI: 0.895–1) (*p*-value 0.003). This means that IL13 is a good biomarker for detecting the severity of allergic conjunctivitis in studied patients. This agrees with Vlaykov et al. who showed that IL13 was correlated with allergic diseases (12). Our study showed that there was a significant positive correlation between total IgE and serum level of IL13 (*r* = 0.809, *P* = 0.0001). This is consistent with Jebur and Saud who showed that there was a positive correlation between IL13 and the total IgE levels (30). There are two limitations in this study that could be addressed in upcoming research. First, lack of follow up of allergic conjunctivitis patients. Second, Lack of prior research studies on the topic. We recommend further study on a wide scale.

## 5 Conclusion

*FOXP3* polymorphism, total IgE and IL13 correlated with the risk and severity of allergic conjunctivitis and may be useful for determining the best line of treatment to allergic conjunctivitis patients.

## Data Availability

The original contributions presented in the study are included in the article/Supplementary Material, further inquiries can be directed to the corresponding author/s.
