# Supplementary material for: Correlation between forkhead box P3 (rs3761548) gene polymorphism and serum interleukin13 as biomarkers of severity in Egyptian allergic conjunctivitis: a retrospective study
Source: Front Allergy. 2024 Sep 25;5:1437600. doi: 10.3389/falgy.2024.1437600 (PMC11461442; doi:10.3389/falgy.2024.1437600)
Supplement: Supplementary file 1 [file Table1.docx]

| SNP ID* | Sequence Position | Accession Number | Identifier | Allele |
| --- | --- | --- | --- | --- |
| rs3761548 | GRCh38.p14 chr X | NC_000023.11 | g.49261784 | G>A, G>C, G>T |

*https://www.ncbi.nlm.nih.gov/snp/rs3761548
